# Tumor Microbiome in Nasopharyngeal Carcinoma and Its Association With Prognosis

**DOI:** 10.3389/fonc.2022.859721

**Published:** 2022-05-23

**Authors:** Guihua Zhong, Wei Wei, Wei Liao, Rong Wang, Yingpeng Peng, Yuling Zhou, Xiaotao Huang, Shiping Xian, Shunli Peng, Zhaoyuan Zhang, Shaoyan Feng, Ye Liu, Haiyu Hong, Yunfei Xia, Yan Yan, Qiaodan Liu, Zhigang Liu

**Affiliations:** ^1^ Cancer Center, The Fifth Affiliated Hospital of Sun Yat-sen University, Zhuhai, China; ^2^ Guangdong Provincial Key Laboratory of Biomedical Imaging, The Fifth Affiliated Hospital of Sun Yat-sen University, Zhuhai, China; ^3^ Department of Otorhinolaryngology, Head and Neck Surgery, The Fifth Affiliated Hospital of Sun Yat-sen University, Zhuhai, China; ^4^ Department of Pathology, The Fifth Affiliated Hospital of Sun Yat-sen University, Zhuhai, China; ^5^ Department of Radiation Oncology, Sun Yat-sen University Cancer Center, State Key Laboratory of Oncology in Southern China, Collaborative Innovation Center for Cancer Medicine, Guangzhou, China; ^6^ Guangdong Provincial Engineering Research Center for Molecular Imaging, The Fifth Affiliated Hospital of Sun Yat-sen University, Zhuhai, China

**Keywords:** nasopharyngeal carcinoma, chronic nasopharyngitis, tumor microbiome, biomarker, progression-free survival

## Abstract

**Introduction:**

Previous studies have reported a close relationship between cancer and microbes, particularly gut and tumor microbiota; however, the presence of tumor microbiome in nasopharyngeal carcinoma (NPC) and its role in the prognosis of NPC remain unclear.

**Methods:**

We collected 64 samples including tissues from 50 patients with NPC (NPC group) and 14 patients with chronic nasopharyngitis (control group) receiver operating characteristics and we applied 16S ribosome RNA gene sequencing of all samples to assess microbiome profiles and immunohistochemistry to detect tumor microbiome in NPC.

**Results:**

Patients in the control group harbored higher species diversity than those in the NPC group; however, the beta diversity was more distinct in the NPC group. In total, three genera with statistically significant differences between the two groups were identified. The area under the receiver operating characteristics (ROC) curve (AUC) was calculated using the relative abundance of these three significant genera, and a value of 0.842 was achieved. Furthermore, *Turicibacter* was confirmed as a potentially independent prognostic factor for NPC patients, and the progression-free survival (PFS) was markedly prolonged in patients with a low relative abundance of *Turicibacter* compared to patients with a high relative abundance of this genus (cutoff: 0.0046, hazard ratio: 5.10, 95% confidence interval: 2.04–12.77, *p* = 0.004).

**Conclusions:**

The present study provided strong evidence of a correlation between tumor microbiome and NPC; the tumor microbiome may be considered a biomarker for early NPC diagnosis. *Turicibacter* potentially served as a independently prognostic indicator for NPC patients.

## Introduction

Nasopharyngeal carcinoma (NPC) is the most common cancer of the head and neck region, and it mainly occurs in East Asian and Southeast Asian populations (1). Moreover, comprehensive treatments based on radiotherapy reveal that the 5-year survival rate of NPC patients is >80% (2). As early symptoms remain inconspicuous, the NPC patients are often initially diagnosed with locally advanced stages, known as stage III or IVa. Induction chemotherapy followed by concurrent chemoradiotherapy for locally advanced NPC can improve the survival rate of patients (3). However, the median overall survival of newly diagnosed metastatic NPC patients was only 29.1 months (4). Therefore, we still need to seek a more effective method for early diagnosis to prolong the survival of NPC patients.

Recent studies have reported a close relationship between cancer and microbes. It is catholically recognized that approximately 17% of human cancers worldwide are caused by microbes (5). Additionally, fecal microbiota plays a crucial role in the development and progression of colorectal cancer (6). This close-related phenomenon is similarly uncovered in other human cancers, particularly between cancer and tumor microbiome. Lung squamous cell carcinoma with TP53 mutations possesses a specific bacterial consortium, and *Acidovorax* had a particularly higher abundance in smoking-related lung cancers (7). Local microbiota in the lung provokes inflammation by activating lung-resident γδ T cells, thereby increasing the development of lung adenocarcinoma. Furthermore, microbes independent of tumor genome can remarkably affect the biological behavior of tumor. A recent study detected 1526 tumor samples including breast, lung, ovary, pancreas, melanoma, bone, and brain tumors, and comprehensively analyzed the tumor microbiome; the results indicated that different tumors exhibited distinct microbiome compositions, which were mainly found in the tumor cells (8). The correlations between intratumor bacteria and certain clinical characteristics of tumors or patient prognosis remain distinctive. To be more exhilarating, a study analyzed the microbiome of 33 different tumor types *via* The Cancer Genome Atlas and certified that microbiomes existed in tumors and blood (9). Therefore, we come to a supposition that the tumor microbiome may play a pivotal role in early diagnosing NPC.

Bacteria can be used as potential biomarkers for tumor diagnosis and prognosis. Colorectal cancer has the most advanced research progress in the diagnosis of gut microbiome. Moreover, a study on the correlation between colorectal cancer and fecal microbiomes by metagenomic profiling analysis indicated that fecal microbiomes could be used for early diagnosis of colorectal cancer (10), such as *peptostreptococcus anerobius*, *parvimonas*, *porphyromonas*, *akkermansia muciniphila*, and *fusobacterium* (11). Meanwhile, a recent study stated that the circulating bacterial DNA in colorectal neoplasia patients reveals a distinct alteration compared to the healthy participants, suggesting that circulating bacterial DNA may prove to be a promising biomarker for colorectal neoplasia (12). The specific oral microbiota in colorectal cancer patients also has a high prediction of diagnosis (13, 14). Besides, the patients with pancreatic head carcinoma reveal a relative abundance in *Haemophilus, Porphyromonas, Leptotrichia*, and *Fusobacterium* can distinguish patients with pancreatic head carcinoma and healthy people (15). The gut microbiome is known to be inextricably interwoven with immunotherapy (16), and its high diversity in metastatic melanoma patients significantly prolonged PFS compared to those with intermediate or low diversity (17). Additionally, tumor microbiome can also act as a prognostic factor for cancer patients, as it influences patient survival in pancreatic cancer independent of therapy (18).

In the present study, we characterized the NPC-associated intratissue microbiome by analyzing 50 tissue samples with NPC and 14 tissue samples with chronic nasopharyngitis. This is the first study to report that tumor microbiome was present in NPC tumor tissues, and may acted as a diagnostic and a prognostic factor in NPC.

## Methods

### Patients and Sampling

In this study, we enrolled 64 subjects including 50 newly diagnosed patients with nasopharyngeal carcinoma (NPC group) and 14 patients with chronic nasopharyngitis (control group) from November 2019 to July 2020. The tissues of all participants were sampled with sterile forceps in a sterile operating room and immediately frozen in liquid nitrogen in sterile cryopreservation tubes before pathological biopsy. In the protocol, patients were included with histologically confirmed nasopharyngeal carcinoma or chronic nasopharyngitis, Eastern Cooperative Oncology Group status of 0–2, at least one site of measurable disease according to the Response Evaluation Criteria in Solid Tumors (RECIST) version 1.1 criteria if coconfirmed nasopharyngeal carcinoma. The exclusion criteria were consuming antibiotics within 2 weeks and having systemic infectious diseases before signing the informed consent form. And in order to explore the relevance between intratumor bacteria and NPC treatments, we performed an average follow-up time of 13.4 months for the NPC participants. Ethical approval was granted by the Ethics Committee of the Fifth Affiliated Hospital of Sun Yat-sen University.

### Immunostaining Assays

Paraffin sections of 15 nasopharyngeal carcinoma and 10 chronic nasopharyngitis tissues from the participants were stained for bacterial lipopolysaccharide (LPS) with Lipopolysaccharide Core (mAb WN1 222-5, 20 HycultBiotech #HM6011, 1:1000 dilution) or no primary antibody (negative control) using the Bond Polymer Refine Detection Kit (Leica Biosystems #DS9800), according to manufacturer’s instructions. Acidic antigens retrieved *via* a 20 min heating step with citrate buffer. Sections were observed using the Leica DMi1 inverted microscope at 40X magnification.

### DNA Extraction, Library Preparation, and Bacterial 16S rRNA Sequencing

Genomic DNA of pathological tissues was extracted and the sample was accurately quantified using Qubit. Thereafter, 30 ng of qualified genomic DNA samples and the corresponding fusion primers (V4F- GTGCCAGCMGCCGCGGTAA and V4R- GGACTACHVGGGTWTCTAAT) were used to configure the PCR reaction system in order to amplify bacterial 16S rRNA V4 fragments. The PCR products were purified using Agencourt AMPure XP beads and dissolved in the Elution Buffer, and then labeled to complete the construction of the library. Agilent 2100 Bioanalyzer was applied to detect the fragment range and concentration of the library. The qualified library was sequenced on the MiSeq platform.

### Bioinformatics Analysis

Raw paired-end 16S rRNA reads (V4 region) were trimmed to at least 15 bp and merged into a single sequence using FLASH v1.2.10 software. Denoising was performed using DADA2 of the QIIME 2 software to obtain amplicon sequence variants (ASVs). Each ASV of 16S rRNA gene sequence was analyzed and highly qualified by SILVA Database v132. All ASVs falling under 0.1% relative abundance in our dataset were excluded to increase the accuracy of the analysis. Veen and Upset diagrams were applied to depict the shared ASVs among the two groups. The microbial community analysis of alpha-diversity was calculated using the following indices: Shannon, inverse Simpson, and Chao1. The beta-diversity index, principal coordinate analysis (PCoA), was measured by Bray-Curtis distance metrics. The specific characterization of the microbiota to distinguish taxonomic types was analyzed *via* a linear discriminant analysis (LDA) effect size (LEfSe) method, and a cladogram was plotted to demonstrate the hierarchy among the discriminative features. Predicted functional genes were categorized into Keyoto Encyclopedia of Genes and Genome (KEGG) to analyze the different metabolic pathways between two groups by the PICRUSt2 tool.

### Statistical Analysis

Statistical analyses were performed using SPSS 25.0 software (IBM SPSS Software, Armonk, New York) and R 4.1. The associations of clinical characteristics between the two different groups were analyzed by Fisher’s test. Alpha-diversity analyses between the two groups were conducted by nonparametric Mann–Whitney tests. Significantly enriched species at the genus level in the two groups were obtained in MaAsLin regression model by MaAslin2 R package after adjusting clinical characteristics. The corresponding genera were concluded and analyzed by the receiver operating characteristics (ROC) curve using Medcalc software v19.6.1 to identify the genera with high predictive ability. The area under the ROC curve (AUC) between two groups was applied to evaluate the diagnostic accuracy of the microbiome. To compare the difference of predicted metabolic pathways, Mann–Whitney tests with Benjamini–Hochberg FDR correction for multiple comparisons was performed, and Q value (False Discovery Rate, FDR) < 0.05 was considered statistically significant. Spearman rank-order correlations were plotted to evaluate associations between significant genera. In addition, Cox proportional hazards model including univariate analysis and multivariate analysis was used to further identify the independent prognostic factors associated with PFS. Eventually, univariate survival analysis of PFS was performed by the Kaplan–Meier method (log-rank test).

## Results

### Tumor Microbiome Exists in NPC and Microbiome Diversities Differ in the NPC and Chronic Nasopharyngitis

**Figure 1:**
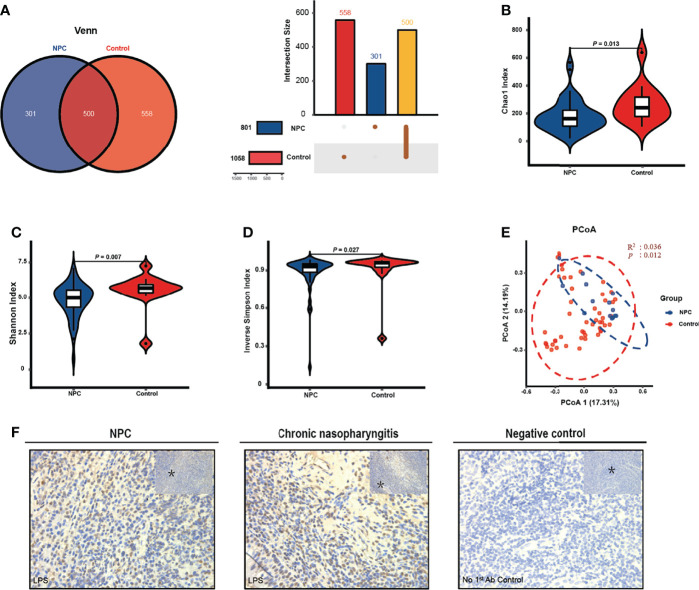
The intratissue microbiome diversity between the nasopharyngeal carcinoma (NPC) group and the control group. **(A)** Venn and UpSet diagrams illustrate shared ASVs in both the NPC and control groups. The alpha diversity in the NPC group and the control group is estimated by Chao1 index **(B)**, Shannon index **(C)**, and inverse Simpson index **(D)**. **(E)** Beta diversity is measured by principal coordinate analysis (PCoA) based on Bray-Curtis distance metrics. **(F)** Representative cores depict the staining of bacterial LPS patterns in NPC and chronic nasopharyngitis. The asterisks indicate the region selected for higher magnification at 40X.

In this study, we collected 64 fresh-frozen tissue specimens from 50 patients with NPC (NPC group) and 14 patients with chronic nasopharyngitis (control group), respectively. The clinical characteristics are listed in **Table 1**. It demonstrated that a significant difference in age was observed between the NPC and control groups. At baseline, the two groups were similar in gender, smoking habits, and alcohol consumption. Firstly, the Venn and UpSet diagrams depicted that 500, 301, and 558 ASVs existed in both groups, merely in the NPC group, and the control group, respectively (**Figure 1A**). To evaluate differences in the microbial structure, we measured the microbial alpha and beta diversities. The alpha-diversity indices including Chao1 index (*p* = 0.013), Shannon index (*p* = 0.007), and Inverse Simpson index (*p* = 0.027) in the NPC group differed significantly from those in the control group (**Figures 1B–D**). Moreover, these indices defined as within-group taxonomic richness and evenness were higher in the control group, indicating higher microbial diversity. Furthermore, a significant difference in beta diversity between the NPC and control groups was also demonstrated by PCoA (**Figure 1E**), suggesting that the intratissue microbial communities exhibit phylogenetic closeness within each group (p = 0.012). Components 1 and components 2 accounted for 17.31% and 14.19% of the variance in PCoA, respectively. To validate the presence of bacteria in NPC and chronic nasopharyngitis, we detected paraffin section slides in 15 of 50 NPC patients and 10 of 14 patients with chronic nasopharyngitis. Additionally, we confirmed the presence of intratissue bacteria in NPC and chronic nasopharyngitis *via* immunohistochemistry with LPS antibody for Gram-negative bacteria (**Figure 1F**).

**Table 1:** Characteristics of the included participants.

Demographic variables	NPC (n = 50)	Control (n = 14)	*p* value
**Age (years)**	50.4 ± 10.8	37.0 ± 13.1	**< 0.001**
**Gender**			0.736
Male	38 (76.0)	10 (71.4)	
Female	12 (24.0)	4 (28.6)	
**Smoker**			0.546
No	33 (66.0)	8 (57.1)	
Yes	17 (34.00)	6 (42.9)	
**Alcohol abuse**			0.673
No	7 (14.0)	1 (7.1)	
Yes	43 (86.0)	13 (92.9)	

The values are presented as the mean ± standard deviation (SD) or n (%) unless otherwise stated. NPC, Patients with nasopharyngeal carcinoma; Control, the control group of patients with chronic nasopharyngitis. p < 0.05 (the bold value) in the table indicates a statistical significance.

### Microbiome Composition in NPC Is Distinct From Chronic Nasopharyngitis

**Figure 2:**
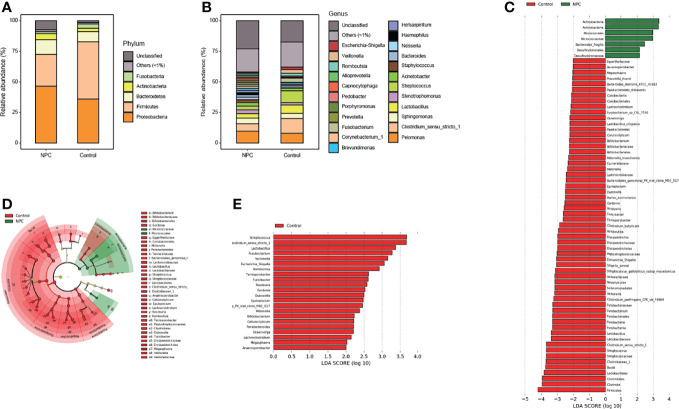
Distinct microbiome composition between the NPC group and the control group. Taxonomic composition in the NPC group and the control group exhibits average relative abundance at the phylum **(A)** and genus **(B)** levels. **(C)** Differential taxa from phylum to species were identified by LEfSe with a meeting LDA significant value (LDA > 2, red color indicating taxa abundant in the control group; green in the NPC group). **(D)** Cladogram shows the differential taxa at all levels from phylum to species. **(E)** Differential taxa at the genus level are represented (LDA > 2, red color indicating taxa enriched in the control group; green in the NPC group).

Considering the differences in diversity, we determined the dissimilarities in the intratissue microbiome composition between the two groups. We calculated the relative abundance of intratissue bacterial composition at the phylum and genus levels. The results showed that the bacterial communities in nasopharyngeal tissues of both the NPC and control groups were dominated by 534 genera in 24 major phyla. The major phyla comprising 92.38% of the total sequences included *Proteobacteria*, *Firmicutes*, *Bacteroidetes*, *Actinobacteria* and *Fusobacteria* (**Figure 2A**). And the top 10 genera with a total of 41.51% relative abundance included *Pelomonas*, *Clostridium_sensu_stricto_1*, *Sphingomonas*, *Streptococcus*, *Lactobacillus*, *Stenotrophomonas*, *Acinetobacter*, *Bacteroides*, *Fusobacterium*, and *Staphylococcus* (**Figure 2B**). To further explore the distinct bacterial taxa responsible for the differences between the two groups, we performed LEfSe analysis at all levels. Sixty-six taxa with differential abundance in the two groups were identified, such as *Actinobacteria* and *Fusobacteria* at the phylum level, *Erysipelotrichia* and *Bacilli* at the class level, *Lactobacillales* and *Fusobacteriales* at the order level, *Micrococcaceae* and *Lactobacillaceae* at the family level, *Clostridium_sensu_stricto_1* and *Streptococcus* at the genus level, and *Lactobacillus_crispatus* and *Millionella_massiliensis* at the species level (**Figures 2C, D**); however, the results illustrated that the distinct bacteria with higher relative abundance in the control group presented higher number than the NPC group. Thus, we plotted all distinct bacteria at the genus level, and it amazingly appeared that all of them were enriched in the control group (**Figure 2E**).

### Diverse Microbiota and Metabolic Functions Are Distinguished Between NPC and Chronic Nasopharyngitis

**Figure 3:**
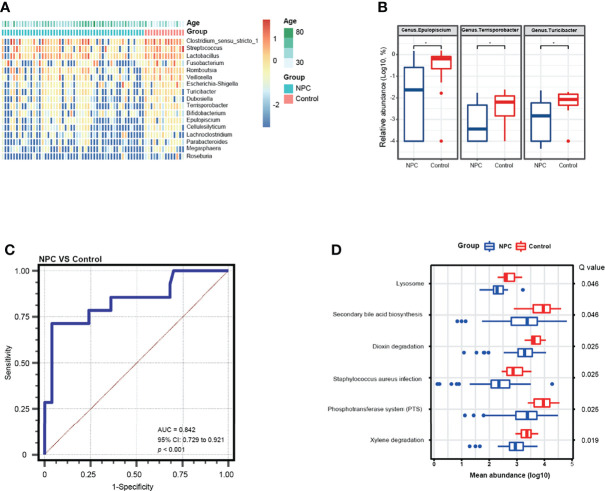
Differential genera and metabolic function pathways associated with NPC and chronic nasopharyngitis. **(A)** A heatmap shows the relative abundance of significant genera between the NPC group and the control group (Mann–Whitney tests, unadjusted *p* value < 0.05). **(B)** Differential genera in relative abundance detected by a MaAsLin model with adjustment for age are exhibited (* means corrected Q value < 0.25). **(C)** ROC curve analysis of differential genera for diagnosis of NPC from chronic nasopharyngitis. **(D)** Distinct metabolic pathways compared between NPC and chronic nasopharyngitis based on KEGG with FDR corrected *p* value (Q value < 0.05).

To further determine the bacterial diversity, we identified 17 bacterial genera that were differentially abundant in the NPC group versus the control group (Mann–Whitney tests, unadjusted for age, *p* < 0.05) (**Figure 3A**). And then we noted that three significant bacterial genera (*Epulopiscium*, *Terrisporobacter*, and *Turicibacter*) were identified by the MaAsLin regression model with consistently higher relative abundance in the control group (**Figure 3B**). Thereafter, we performed a receiver operating characteristics analysis by the logistic regression model to evaluate the capability of these three genera in discriminating NPC from chronic nasopharyngitis; we found that the combination of *Epulopiscium*, *Terrisporobacter*, and *Turicibacter* resulted in an AUC of 0.842 after adjustment for age (**Figure 3C**). Furthermore, we analyzed the abundance of KEGG pathways. The significant pathways with Q value corrected by FDR including xylene degradation, phosphotransferase system (PTS), *Staphylococcus aureus* infection, dioxin degradation, secondary bile acid biosynthesis, and lysosome (Q value < 0.05), were all enriched in the control group (**Figure 3D**).

### Correlation Analysis of Significantly Bacterial Genera Shows Different Patterns in the Two Groups

**Figure 4:**
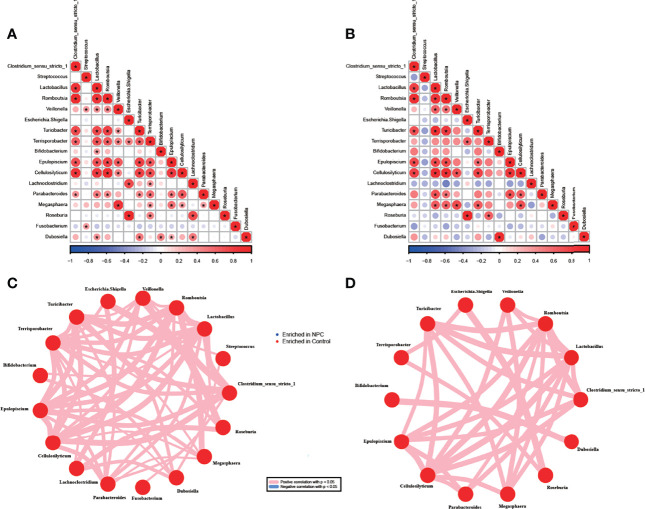
Correlations between genera in the NPC group and the control group. Correlation among 17 significant genera with unadjusted *p* value < 0.05 in the NPC group **(A)** and the control group **(B)** (*correlation *p* value < 0.05, red color indicating taxa enriched in the control group; blue in the NPC group). Distinct patterns of significant genera in the NPC group **(C)** and the control group **(D)** are plotted by network plot based on statistically significant correlations (correlation *p* value < 0.05).

To further understand the potential interaction among differentially abundant bacterial genera, we performed Spearman correlation analysis in NPC and chronic nasopharyngitis. A significant positive correlation was found in 53 genera pairs in the NPC group (*p* < 0.05, **Figures 4A, C**) and 31 genera pairs in the control group (*p* < 0.05, **Figures 4B, D**). Although all the correlations between genera were positive, great distinctions in the correlation pattern of differentially abundant bacterial genera between the NPC group and the control group were apparent, and the NPC group displayed a more intricate correlation pattern. The most abundant genus *Clostridium_sensu_stricto_1* exhibited a positive correlation with seven genera in the NPC group and five genera in the control group, respectively. *Terrisporobacter* revealed highest interaction in the NPC group, and correlated with *Clostridium_sensu_stricto_1*, *Lactobacillus*, *Romboutsia*, *Veillonella*, *Escherichia-Shigella*, *Turicibacter*, *Epulopiscium*, *Cellulosilyticum*, *Lachnoclostridium*, *Parabacteroides*, *Megasphaera*, and *Roseburia*. *Lactobacillus*, *Romboutsia*, and *Cellulosilyticum* showed the highest interactions in the control group, which were all correlated with eight bacterial genera. Furthermore, some strong correlations between the intratissue genera in the NPC group, such as *Lachnoclostridium* and *Roseburia*, were not shown in the control group. Similarly, some strong correlations of intratissue genera in the control group, such as *Megasphaera* and *Lactobacillus*, were not observed in the NPC group.

### Tumor Bacteria Is Associated With PFS of NPC Patients

**Figure 5:**
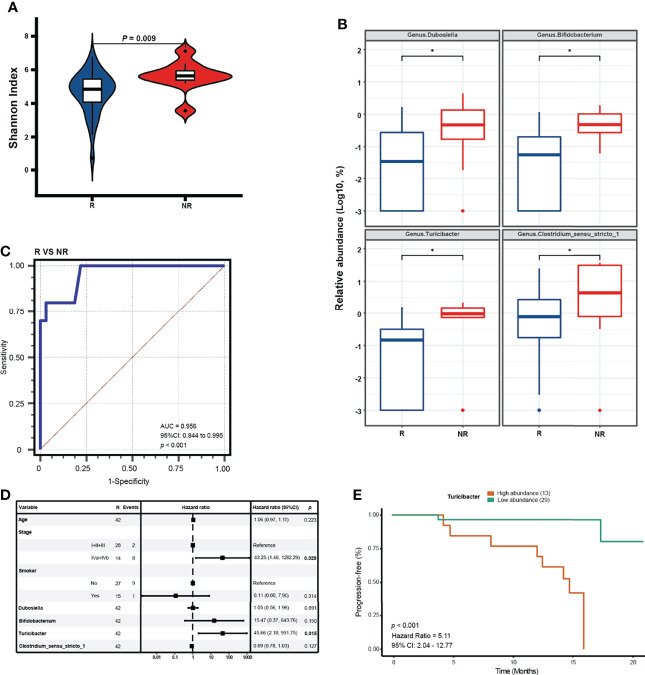
Differential genera serving as prognostic factors for NPC. **(A)** The alpha diversity in the NR and R groups is estimated by Shannon index. **(B)** Significant genera in relative abundance detected by a MaAsLin model with adjustment for age * means (corrected Q value < 0.25). **(C)** ROC curve analysis of differential genera for diagnosis of R from NR for NPC treatments. **(D)** Forest plot reveals the selected covariates on clinical characteristics and significant genera tested by multivariate COX regression analysis. **(E)** Kaplan-Meier (K-M) plot of the PFS by Log-rank test in patients with high relative abundance and low relative abundance of *Turicibacter* (cutoff: 0.0046).

We retrospectively evaluated the responses of patients in the NPC group to standard treatments. We followed up with 42 patients among the 50 NPC patients for an average time of 13.4 months. After standard treatments including chemotherapy and radiotherapy, 32 patients were evaluated for partial response or stable disease, who were defined as the R group, whereas 10 patients with disease progression after the first evaluation were defined as the NR group. The clinical characteristics revealed no significance in age, gender, smoking habits, and alcohol consumption; however, the clinical stage significantly differed between the R and NR groups (**Table 2**). The alpha diversity analysis demonstrated that the NR group suffered higher Shannon index than the R group, indicating higher microbial diversity (**Figure 5A**). Then we noted that four significant bacterial genera (*Dubosiella*, *Bifidobacterium*, *Turicibacter*, and *Clostridium_sensu_stricto_1*) were identified by the MaAsLin regression model after adjusting for clinical stage and age to obtain a more accurate prediction result (**Figure 5B**). We found that the combination of these four differently abundant genera resulted in an AUC of 0.956 after adjustment for clinical stage and age (**Figure 5C**). Then, we conducted a univariate Cox analysis between the relative abundance of these 4 genera and PFS, and found that the significant correlations between them were confirmed (p < 0.05) (**Table 2**). Further, the multivariate analysis revealed that bacterial genus *Turicibacter*, instead of three other significant genera, was confirmed as an independent risk factor for PFS (hazard ratio: 45.66, 95% confidence interval: 2.10–991.75, p = 0.015) (**Figure 5D**). In order to further confirm the correlation between tumor microbiome and different clinical stages, we analyzed the alpha and beta diversities in different stages which were divided into two groups including I + II + III group and IVa + IVb group. The results showed that the two groups of clinical stages presented no significant difference in alpha and beta diversities, and demonstrated that tumor microbiome in relatively early stages and relatively advanced stages was basically consistent. The PFS was significantly prolonged in patients with low relative abundance of *Turicibacter* compared to patients with high relative abundance (cutoff: 0.0046, hazard ratio: 5.10, 95% confidence interval: 2.04 –12.77, p = 0.004) (**Figure 5E**).

**Table 2:** Characteristics and univariate analysis of PFS in the participants with NPC.

Covariates	R (n = 32)	NR (n = 10)	*p* value	Univariate analysis	
				HR (95% CI)	*p* value
**Age (years)**	48.8 ± 7.4	57.7 ± 13.3	0.068	1.087 (1.01, 1.12)	**0.023**
**Gender**			0.606		
Male	24 (75.0)	6 (60.0)		Reference	
Female	8 (25.0)	4 (40.0)		1.34 (0.37, 4.77)	0.656
**Smoker**			0.117		
No	18 (56.2)	9 (90.0)		Reference	
Yes	14 (43.8)	1 (10.0)		0.10 (0.01, 0.85)	**0.035**
**Alcohol abuse**			1.000		
No	28 (87.5)	9 (90.0)		Reference	
Yes	4 (12.5)	1 (10.0)		0.60 (0.07, 4.90)	0.635
**Clinical stage**			**0.001**		
I + II + III	26 (81.2)	2 (20.0)		Reference	
IVa + IVb	6 (18.8)	8 (80.0)		7.66 (1.61, 36.43)	**0.011**
**Dubosiella**				1.69 (1.16, 2.48)	**0.007**
**Bifidobacterium**				3.78 (1.45, 9.87)	**0.006**
**Turicibacter**				5.11 (2.04, 12.77)	**<0.001**
**Clostridium_sensu_stricto_1**				1.06 (1.01, 1.11)	**0.012**

The values are presented as the mean ± standard deviation (SD) or n (%) unless otherwise stated.

R, Patients without progressive disease after standard treatments; NR, Patients with progressive disease after standard treatments. p < 0.05 (the bold values) in the table indicates a statistical significance.

## Discussion

In the present study, we initially characterized the intratissue microbiome associated with NPC and chronic nasopharyngitis and assessed the ability of intratumor bacteria in predicting patient prognosis. We found that the tissue-resident bacteria existed in the tumor and chronic nasopharyngitis detected *via* immunohistochemistry. Our results showed that the microbiota distinctions between NPC and chronic nasopharyngitis were evident and taxonomic richness and evenness varied consistently as determined by Chao1 index, Shannon index, and inverse Simpson index. The proportion of major bacteria in chronic nasopharyngitis was larger than that in NPC, as visualized by intratissue microbiome composition of NPC and chronic nasopharyngitis. We then identified differentially abundant bacterial genera discriminating NPC from chronic nasopharyngitis with an AUC value of 0.842. Additionally, the uncommon phenotype of microbiome in NPC revealed more correlations among the intratissue genera, thereby indicating a more complex microbial ecology than chronic nasopharyngitis. Eventually, multivariate analysis revealed that the specific intratumor bacterial genus *Turicibacter* was an independent prognostic factor of NPC patients, which was further confirmed by no significant difference between different clinical stages and tumor microbiome.

The intratissue microbiota has been validated to exert important effects in various tumors and normal tissues (8, 19); however, the presence of tumor microbiome in NPC and whether it could serve as diagnostic biomarker remain unclear. Thus, we collected NPC tissues and chronic nasopharyngitis tissues as control group, for chronic nasopharyngitis shares the same presentation symptoms with NPC and is the common differential disease (20). The present study provided evidence of intratissue microbiome in NPC and chronic nasopharyngitis. Moreover, bacterial diversity in the gut, defined as taxonomic richness and evenness, plays a crucial role in numerous diseases, particularly in obesity, inflammatory bowel disease, and cancer (7, 21–23). Although the diversity of gut microbiome influences the immunotherapy responses, the diversity of the tumor microbiome has been proven to influence survival in pancreatic cancer independent of therapy (16, 18). Our findings suggest that the diversity in NPC was lower than in chronic nasopharyngitis, and the proportion of major bacterial genera in chronic nasopharyngitis was larger than that in NPC. Similarly, though the result of beta-diversity analysis was significantly different between the two groups, the cluster of NPC group and chronic nasopharyngitis group was obviously indistinguishable, thereby indicating major difference in the rare or less abundant bacteria. We identified three differentially abundant bacterial genera *Epulopiscium*, *Terrisporobacter*, and *Turicibacter*, which were not the top 10 abundant genera in each group. Thus, we put forward a hypothesis that the bacterial genera with medium abundance in NPC played a more important role than those in chronic nasopharyngitis and perhaps influenced the development and progression of NPC.

To the best of our knowledge, tumor microenvironment is complex. Recent studies have identified the tissue-resided microbiome in tumors or other human tissues (8, 19, 24). However, the interaction of the intratissue microbiome still remains vague. Our results show that NPC presents more complex interactions among 17 differentially abundant genera than chronic nasopharyngitis. Chronic nasopharyngitis is mainly caused by microbial infection (25). It was not surprising given that chronic nasopharyngitis had higher microbiome diversity than NPC whereas NPC presented a more complicated microbiome interaction which might be caused by the tumor microenvironment. We also found that several metabolic pathways were enriched in chronic nasopharyngitis compared to NPC, such as *Staphylococcus aureus* infection, dioxin degradation, and xylene degradation. *Staphylococcus aureus* infection reveals pathogenicity in chronic nasopharyngitis (26). Dioxin and xylene Group 1 and Group 3 may increase the risk of cancer (27, 28). Though there was no direct research evidence of a relationship between dioxin and xylene and NPC, our study predicted that accumulation of dioxin and xylene caused by reduced degradation capacity was associated with NPC. However, further research is needed to confirm this.

Gut microbiome composition is closely associated with the outcome of immunotherapy or chemotherapy in various tumors (18, 29). In a recent study, intratumor bacterium *Gammaproteobacterial* was demonstrated to induce gemcitabine resistance (30). In esophageal cancer tissue, *Fusobacterium nucleatum* was identified as a potential prognostic biomarker for survival (31). The present study revealed that *Turicibacter* acted as an independent prognostic factor of PFS in NPC patients despite the therapeutic methods used. That means intratumor bacteria may act as predictive biomarkers for the prognosis of NPC patients in the future.

Though our study presented the microbiome profiles in NPC and chronic nasopharyngitis, it has certain limitations. Firstly, more participants were required to enroll to increase the credibility of the conclusions and our study lacked a validation cohort. Secondly, for its retrospective nature, our findings could not determine the causality between intratumor microbiome and NPC. For instance, whether the complicated interactions of intratumor microbiome in NPC promote tumorigenesis or the tumorigenesis could induce complicated interactions of intratumor microbiome remains unclear. More experimental evidences are essential to corroborate these issues in the future. Additional studies to address the mechanism by which intratumor microbiome affects the treatment responses and to determine whether different bacteria affect various treatments might also be considered. Thirdly, we only followed up with an average time of 13.4 months in the present study. Due to the fact that our NPC patients were major in locally advanced or advanced stage, their progression-free survival time may be relatively short. Thus, we followed up with the above time to initially investigate the relationship between PFS and tumor microbiome. The PFS and overall survival time of the patients and its correlation with tumor microbiome need to be further explored with longer follow-up time and larger participants in the future

In conclusion, the present study is the first to confirm the presence of intratissue microbiome in NPC and chronic nasopharyngitis. The intratissue bacterium *Turicibacter* was confirmed as a diagnostic biomarker to discriminate NPC from chronic nasopharyngitis as well as a prognostic predictor of PFS in NPC patients. This study shows the potential ability of tumor microbiome diagnosing nasopharyngeal carcinoma and paves the way for the potential diagnostic exploration of the microbiome to find a noninvasive method for early diagnosis, and to predict the prognosis of nasopharyngeal carcinoma, thereby helping oncologists make personalized treatments decisions for NPC patients.

## Data Availability Statement

The datasets presented in this study can be found in online repositories. The names of the repository/repositories and accession number(s) can be found below: NCBI Sequence Read Archive with BioProject ID PRJNA775826 (https://www.ncbi.nlm.nih.gov/sra/PRJNA775826).

## Ethics Statement

The studies involving human participants were reviewed and approved by Ethics Committee of the Fifth Affiliated Hospital of Sun Yat-sen University (protocol code ZDWY[2020]LunziNo. (K67-1) and approval on 2020.09.24). The patients/participants provided their written informed consent to participate in this study.

## Author Contributions

ZL, QL, and YY proposed the conception, designed this study, wrote and edited the manuscript, and jointly supervised the project. GZ, WW, and WL collected and analyzed data, prepared the figures, tables and manuscript, and contributed equally to this work. RW, YP, YZ, XH, and SX contributed to the collection of clinical samples, data interpretation, and manuscript editing. SP and ZZ contributed to manuscript editing. SF, YL, HH, and YX contributed to data interpretation and manuscript editing. All authors involved in the final approval of the manuscript.

## Funding

This study was supported by grants from the following projects: the Key Training Project for Young Teachers of Sun Yat-sen University Grant (19kyzd07), the Department of Science and Technology of Guangdong Province to the Guangdong Provincial Key Laboratory of Biomedical Imaging (2018B030322006) and the Innovation Research Team for Basic and Clinical Studies on Chronic Liver Diseases of 2018 High-Level Health Teams of Zhuhai.

## Conflict of Interest

The authors declare that the research was conducted in the absence of any commercial or financial relationships that could be construed as a potential conflict of interest.

## Publisher’s Note

All claims expressed in this article are solely those of the authors and do not necessarily represent those of their affiliated organizations, or those of the publisher, the editors and the reviewers. Any product that may be evaluated in this article, or claim that may be made by its manufacturer, is not guaranteed or endorsed by the publisher.

## Abbreviations

NPC, Nasopharyngeal carcinoma; AUC, area under the ROC curve; LPS, Lipopolysaccharide; ASVs, Amplicon sequence variants; PCoA, Principal coordinate analysis; LDA, Linear discriminant analysis; LEfSe, Linear discriminant analysis effect size; KEGG, Keyoto Encyclopedia of Genes and Genome; ROC, receiver operating characteristics; FDR, False Discovery Rate.
