# Supplementary figures and images for: Tumor Microbiome in Nasopharyngeal Carcinoma and Its Association With Prognosis
Source: Front Oncol. 2022 May 23;12:859721. doi: 10.3389/fonc.2022.859721 (PMC9168217; doi:10.3389/fonc.2022.859721)

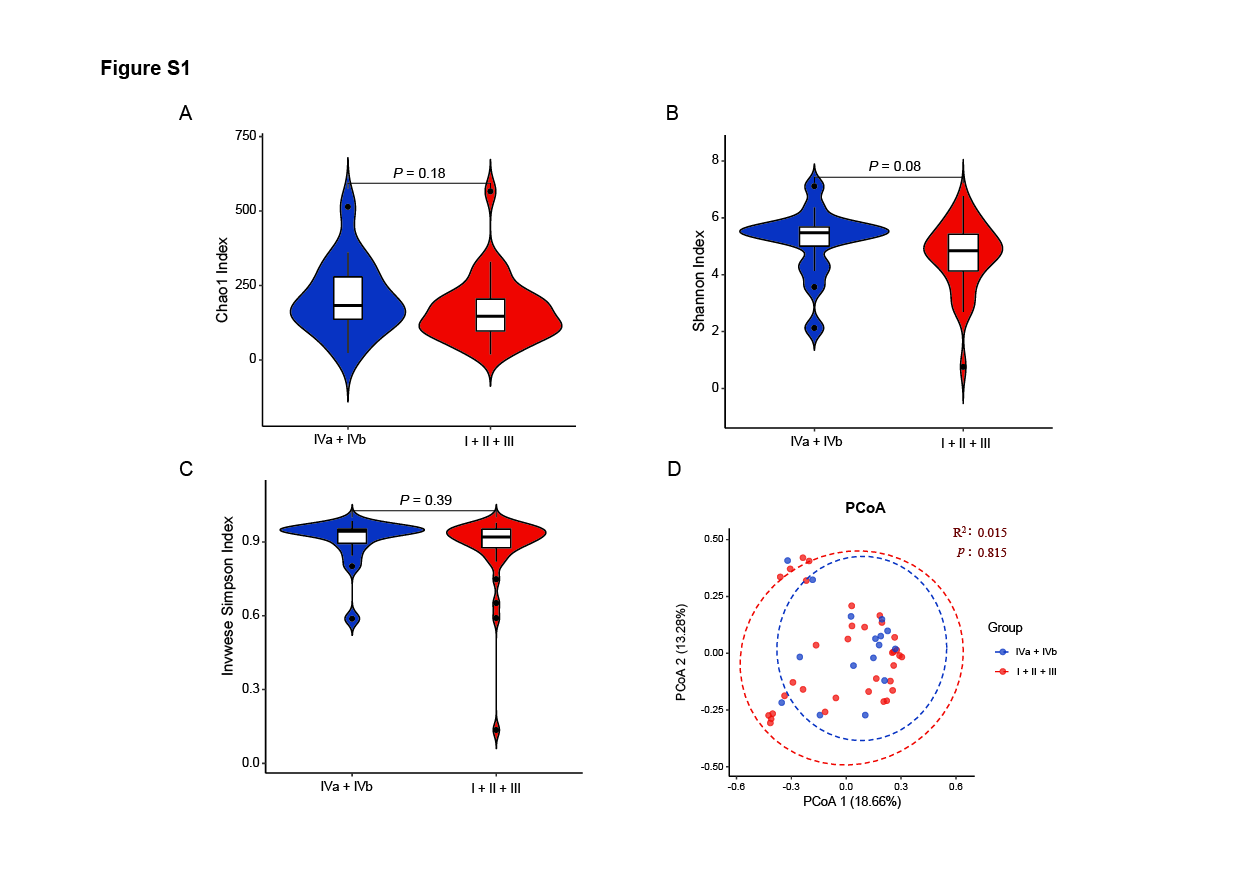

Supplement: Supplementary file 1 [file Image_1.tif]
